# Effect of Added Carbohydrates on Glycemic and Insulin Responses to Children’s Milk Products

**DOI:** 10.3390/nu5010023

**Published:** 2013-01-10

**Authors:** Jennie Brand-Miller, Fiona Atkinson, Angela Rowan

**Affiliations:** 1 School of Molecular Bioscience and Boden Institute, University of Sydney, Sydney, NSW 2006, Australia; E-Mail: fiona.atkinson@sydney.edu.au; 2 Fonterra Research Centre, Private Bag 11029, Palmerston North 4442, New Zealand; E-Mail: Angela.Rowan@fonterra.com

**Keywords:** milk, carbohydrate, glycemia, insulinemia, glycemic index

## Abstract

Powdered milk products for children (*Growing Up Milk Powders* or GUMPs) containing added carbohydrates such as glucose and sucrose are now well established in parts of Asia. We surveyed GUMPs in Malaysia and Indonesia to determine the content of added carbohydrates. The ingredient lists and nutrition information panels were used to calculate the percentage of declared carbohydrates contributed by added carbohydrates and a subset of seven products was tested for their glycemic index (GI) and insulin responses in healthy adults. The glycemic load for each product was calculated. In total, 58 products (*n* = 24 in Malaysia and *n* = 34 in Indonesia) were surveyed. Added carbohydrate content (excluding fibre) ranged from 0 to 21.5 g per serve. Milk powders without added sources of carbohydrate had similar GI values to standard liquid whole milk. Products containing maltodextrins, corn or glucose syrups increased the GI by more than 2-fold, and glycemic load (GL) by 7-fold compared to milk powders with no added carbohydrates. Insulin responses were significantly but not strongly correlated with glucose responses (*r* = 0.32, *p* < 0.006). Children’s milk powders containing higher levels of added carbohydrate ingredients elicit higher glucose and insulin responses than liquid or powdered whole milk.

## 1. Introduction

Milk and dairy products are staples of children’s diets in countries with a long tradition of dairying, and increasingly in countries of Asia where dairying is still largely absent. In western countries, dairy products are recommended as one of the 4–5 food groups in official dietary guidelines. Milk contributes high quality protein and significant quantities of micronutrients, including calcium, riboflavin, vitamin A and zinc. More recently milk is being used as a delivery vehicle for other nutrients such as vitamin D and iron. In Asian countries, a new category of high value milk products for children known as *Growing Up Milk Powders* (GUMPs) has become established in a short timeframe [1]. They are perceived by health professionals and carers as a valuable supplement to children’s diets to help ensure they obtain adequate nutrition, particularly if the diet is generally poor.

Unfortunately, there is a growing trend for manufacturers to incorporate additional carbohydrates such as sucrose, maltodextrins and corn or glucose syrups. Some of these ingredients may be useful in small amounts to increase palatability and encourage consumption of an otherwise bland food. But many products contain high levels of added carbohydrates without detectable sweetness that dilute the nutritional value of the milk. The additional calories and higher energy density may contribute to the trend of increasing overweight and obesity among children in Asia [2]. Discretionary calories are also more concerning if they have the capacity to stimulate excessive postprandial hyperglycaemia and insulinemia. Postprandial glycaemia has dose-related harmful effects that increase the risk of obesity, type 2 diabetes and coronary heart disease [3,4,5].

The current research was therefore undertaken to determine the extent to which additional carbohydrates were being incorporated into GUMPs for children in two Asian countries (Malaysia and Indonesia), and to assess the impact of a selection of these products on blood glucose and insulin responses. The glycemic index (GI) and insulin index (II) were determined according to standardised protocols. 

## 2. Experimental Section

A total of 58 GUMPs (24 from Malaysia and 34 from Indonesia) were surveyed for total and added carbohydrate content. The ingredient lists and nutrition information panels were used to calculate the percentage of declared carbohydrates coming from added sources of sugars and refined carbohydrate ingredients, excluding dietary fiber ingredients (e.g., inulin). In Indonesia, but not Malaysia, the total carbohydrate declaration on the Nutrition Information Panel generally includes fiber. The carbohydrate, protein and fat content of standard full cream and non-fat milk powder were used for reference [6].

A sub-set of 7 products (Table 1) was selected to be representative of those available with a range of added carbohydrate contents. They included GUMPs with no added carbohydrates and those with high levels of mono- or disaccharides and/or maltodextrins (*i.e.*, short chain glucose polymers). Equivalent carbohydrate portions (25 g) were tested for their GI according to the International Standards Organisation methodology [7]. All experimental procedures were conducted in accordance with ethical research in human subjects, and the protocol was approved by the Human Research Ethics Committee of the University of Sydney.

Subjects were recruited from the staff and student population of the University of Sydney. Eleven healthy adults (mean ± SD, age 27.8 ± 4.4 years; BMI 22.2 ± 2.2 kg/m^2^) consumed each of the test products on separate occasions. Each was consumed as reconstituted milk powder prepared according to the manufacturer’s instructions and containing 25 g of available carbohydrate per serving (Table 1). 

**Table 1:** Energy and macronutrient composition of the foods tested per 25 g carbohydrate portion, normal recommended serving size and carbohydrate content of normal serving size.

Product	Energy (kJ)	Total CHO ^1^ (g)	Added CHO ^1^ (g)	Fibre (g)	Protein (g)	Fat (g)	Serve Size Tested ^2^ (g)	Normal Serve Size ^3^ (g)	CHO ^1^ per Normal Serve ^4^ (g)
*1 ^a^*	152.1	25.0	15.3	0.8	4.3	4.0	36	40	28.0
*2 ^b^*	176.0	25.0	13.0	0.0	5.9	6.0	41	44	27.0
*3 ^a^*	296.7	25.0	0.0	1.4	15.7	14.3	64	28	10.9
*4 ^b^*	166.5	25.0	14.2	1.7	5.8	5.8	38	46	29.9
*5 ^a^*	179.7	25.0	13.5	1.3	8.1	5.3	42	40	23.6
*6 ^b^*	163.5	25.0	11.5	1.0	6.7	4.8	38	40	26.0
*7 ^a^*	186.4	25.0	13.8	1.3	7.0	6.4	42	40	23.6
*Liquid whole cow’s milk ^c^*	1305.9	25.0	0.0	0.0	15.8	16.8	508 mL	250 mL	12.3

^1^ Carbohydrate; ^2^ Actual serving size per 25 g available carbohydrate portion; ^3^ Normal serving size according to manufacturer as at May 2011; ^4^ Normal carbohydrate content of manufacturer’s serving; ^a^ Country of origin is Malaysia; ^b^ Country of origin is Indonesia; ^c^ Country of origin is Australia.

Liquid whole cow’s milk was tested for comparison. The reference food, glucose (Glucodin^®^ powder, Boots Health Care Company, Sydney, Australia), was tested on three separate occasions. Each test was completed on a separate morning with at least one day between sessions. On arrival at the metabolic kitchen, two fasting capillary blood samples (approximately 0.8 mL) were taken within 5 min from warmed fingers and collected into Eppendorf tubes containing heparin. The test food or reference food was then consumed with an additional 250 mL of glass of water within 12 min. Further blood samples were taken at 15, 30, 45, 60, 90 and 120 min. Blood samples were centrifuged and the plasma layer collected and stored for glucose analysis on the same day or at −20 °C for insulin analysis within 1 month.

Each plasma sample was analysed in duplicate using a glucose hexokinase assay (Roche Diagnostic Systems, Sydney, Australia) and an automated centrifugal spectrophotometric analyser (Roche/Hitachi 912, Boehringer Mannheim GmbH, Ingelheim am Rhein, Germany). Plasma glucose levels were plotted against time to generate a curve from which the incremental area under the curve was calculated. The GI and glycemic load (GL) for each product were calculated using the following equations:

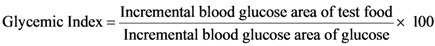


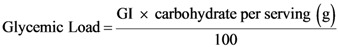



The insulin concentration in each plasma sample was analysed using a solid-phase antibody-coated tube radioimmunoassay kit (Coat-A-Count^®^ Insulin RIA kit, Diagnostic Products Corporation, Los Angeles, CA, USA) with internal controls. The two fasting blood samples were averaged to provide one baseline insulin concentration. The incremental area under the insulin response curves (iAUC) were calculated according to the trapezoidal method and any area under the baseline (fasting value) was ignored. An insulin index (II) value was then calculated by dividing the iAUC for each food by the average iAUC for the reference food and multiplying by 100. The results were analysed using a general linear model (ANOVA) for iAUC with treatment or food and time as fixed factors and subject as a random factor. Results are expressed as means ± SD or mean SEM ± SEM as indicated.

## 3. Results

Supplementary Table S1 shows the range of added carbohydrate content in the products surveyed. On average, GUMPs contained (mean ± SD) 59.6 ± 7.5 g/100 g total carbohydrates, ranging from 38.9 to 71.0 g per 100 g. Added carbohydrate content ranged from 0 to 42.8 g/100 g, or 0 to 21.5 g per serve according to manufacturer’s instructions (mean ± SD: 11.0 ± 11.7 g/11.0 ± 6.2 g). Only 4 products of the 58 products (~7%) contained no additional carbohydrate. The most common source of added carbohydrate was sucrose or cane sugar, followed by glucose syrup solids, corn syrup solids and maltodextrins (Supplementary Table S1). 

**Figure 1:**
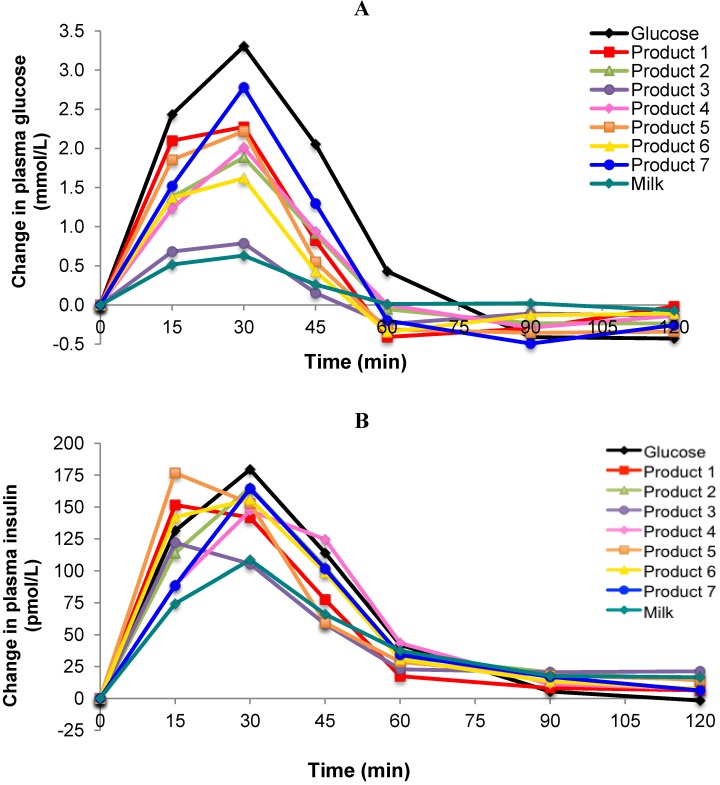
(**A**) Incremental changes in plasma glucose after consumption of 25 g carbohydrate portions of 7 brands of *Growing Up Milk Powders* compared to the reference food (glucose) and regular milk; (**B**) Incremental changes in plasma insulin to 25 g carbohydrate portions of 7 brands of *Growing Up Milk Powders* compared to the reference food (glucose) and regular milk.

**Figure 2:**
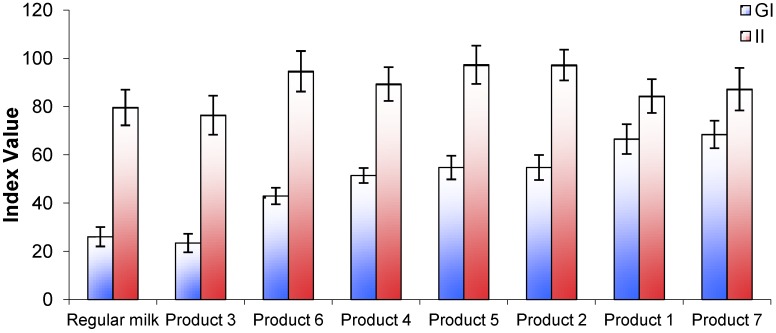
Glycemic index (GI) and insulin index (II) values for 7 brands of *Growing Up Milk Powders* compared with regular fluid milk.

**Figure 3:**
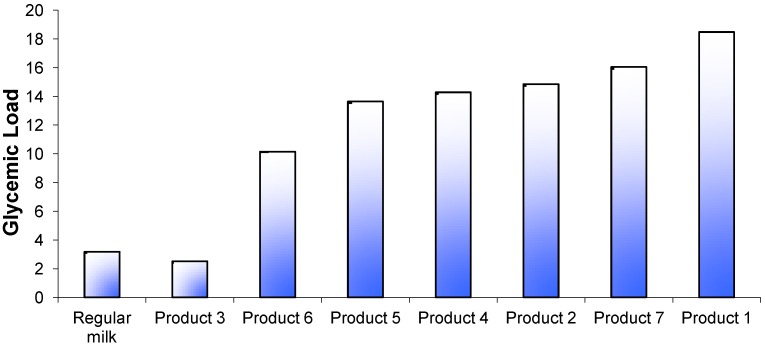
Glycemic load values for 7 brands of *Growing Up Milk Powders* compared with regular fluid milk. The calculation was based on standard serving sizes recommended by the manufacturer (Table 1) and carbohydrate content according to manufacturer’s data.

The incremental plasma glucose response curves for the test foods, the reference food (glucose solution) and regular fluid milk are shown in Figure 1A. One product (Product 3) produced a markedly lower glycemic response similar to that of regular milk. Another product (Product 7) produced a late peak response at 30 min from the start of consumption but the majority appeared to peak at a point between 15 and 30 min. The GI values ranged from 23 ± 4 for Product 3, to 68 ± 6 for Product 7 (Figure 2). In pairwise comparisons, the GI value of Product 3 was significantly lower than Products 2, 4, and 6 (*p* < 0.001) and Product 5 (*p* < 0.01). The calculated GL for each product ranged from 3 to 18 (Figure 3).

All products tested produced insulin response curves that paralleled the rise and fall in glucose responses. On an individual subject basis, insulin AUC and glucose AUC were significantly but not strongly correlated (*r* = 0.32, *p* = 0.006, *n* = 71). Overall, the lowest insulin response was seen for Product 3 (Figure 2), although all insulin responses were relatively high compared with their respective glycemic response. 

## 4. Discussion

This survey of a popular, new category of dairy product in Asian markets (“growing up milk powders” or GUMPs) revealed a wide range of added carbohydrate content. Among 58 products marketed in Indonesia and Malaysia, over 90% contained at least some added carbohydrate, with the highest being 43% by weight added carbohydrate. Only a minority (7%), all from the same manufacturer, contained no additional carbohydrate. Glucose or corn syrup solids, maltodextrins, sucrose, lactose and fructose were the most common additives. Because carbohydrates vary in their potential to raise postprandial glycemia and insulinemia, we measured metabolic responses to a selection of GUMPs, representing both the lowest and highest levels of added carbohydrate. Our findings indicated that two of the seven products had GI values of 60–70, a range similar to sugar-sweetened soft drinks, and 2-fold higher than the products without added carbohydrates. The combination of relatively high GI score and high carbohydrate content per single serving (as directed by manufacturer) meant that one product had a GL 7-fold higher than plain milk. Products without added sources of carbohydrate had similar GI and insulin responses to standard liquid milk. 

Our analysis of insulin responses to GUMPs indicated, as expected, that all GUMP_S_ elicit disproportionately higher insulin responses compared with their GI values. This observation is consistent with findings for protein-rich foods and milk products in general [8,9,10]. Protein and some amino acids stimulate the incretin axis (GIP, GLP-1) and insulin release from the beta-cells, and that the combination of protein and carbohydrate together may be synergistic [11]. Cow’s milk is unique in that it naturally contains a relatively high ratio of protein to carbohydrate (1:1.3) compared with other foods. The proportions might be conducive to programmed anabolism in growing mammals. However, additional carbohydrates dilute the protein fraction (reduce% energy as protein), an effect that might reduce satiety [12] and encourage accretion of body fat mass rather than lean muscle mass [13].

Plain milk is a low GI/GL food with a composition that may be protective against the development of insulin resistance and chronic disease [14]. However, Sharma *et al.* [15,16] recently reported that higher intake of added sugars, including those in dairy foods and beverages, was significantly associated with cardiovascular risk factors in overweight African-American children. There was no association with the natural sugars in dairy products. In observational studies in adult populations, diets containing higher quantities of refined sugars and refined starches have been associated with higher risk of obesity and type 2 diabetes [5,17]. Both quantity and quality of carbohydrate are relevant. Specifically, diets with a high GL (carbohydrate content per serving × GI/100) have been linked with higher relative risk of chronic disease, including cardiovascular disease, diabetes, stroke and some forms of cancer. In prospective studies in children and adolescents, higher total carbohydrate intake and higher GL, have been linked to increased BMI and waist circumference [18], and poor visual acuity [19]. Conversely, carbohydrates of high quality, such as fruit, vegetables and low fat dairy products generally have a low GI and play a role in lowering BMI and waist circumference. In countries experiencing periods of rapid economic development such as those in South East Asia, dietary changes similar to those seen in Western populations are observed along with changes in the prevalence of overweight and nutrition-related diseases such as cardiovascular disease and type 2 diabetes [20]. 

A limitation of our study is that we tested young adults, but the resulting GI values are likely to predict the magnitude and ranking of responses in young children. Randomized controlled trials in children also provide evidence of a beneficial effect of lower dietary GL. The DioGenes study undertaken in families with children aged 5–18 years, found an obesity-protective effect of a diet with a low GI and high protein composition for prevention of weight regain after weight loss [21]. Others have reported that children consuming a low GI or a low GL breakfast had either lower ratings of hunger [22] or lower energy intake at a subsequent meal [23].

## 5. Conclusions

In conclusion, our study provides evidence that milk products targeted at very young children may contain high levels of added carbohydrates that increase caloric density and contribute to higher postprandial glycemia. In Asian countries where GUMPs now make up a considerable portion of the diet of young children [1], there is the potential for the risks to outweigh the benefits if manufacturers do not take a responsible approach in formulation.

## Supplementary Files

Supplementary File 1 Supplementray Information (PDF, 75 KB)
