# Supplementary material for: Effect of Added Carbohydrates on Glycemic and Insulin Responses to Children’s Milk Products
Source: Nutrients. 2013 Jan 10;5(1):23–31. doi: 10.3390/nu5010023 (PMC3571636; doi:10.3390/nu5010023)
Supplement: Supplementary File 1 — Supplementray Information (PDF, 75 KB) [file nutrients-05-00023-s001.pdf]

Supplementray Information

Table S1. Total and calculated added carbohydrate content of 58 *Growing Up Milk Powder* products available in Malaysia and Indonesia.

Manufacturer	Product name	Total CHO per 100 g	Calculated Added CHO per 100 g	Calculated Added CHO per serve [1,2], g	Form of added sugars [3]	Country of origin
Abbott	Gain Plus Advance Plain (1+)	50.3	13.9	5.0	Lactose, sucrose	Indonesia
Abbott	Gain Plus Eye Q Step 3 (Vanilla) (1+)	49.9	15.7	5.0	Lactose, sucrose	Malaysia
Abbott	Gain School Advance Vanilla (3+)	58.0	19.4	7.0	Lactose, sucrose, corn syrup solid	Indonesia
Danone Dumex	Dumex Dugro 1–3 years (1+)	70.2	43	17.2	Glucose syrup solids, lactose, sucrose	Malaysia
Danone Dumex	Dumex Mamil Gold Step 3 (1+)	70.1	42.8	17.1	Lactose, glucose syrup solids, sucrose	Malaysia
Danone Dumex	Dumex Dugro 3–6 years (3+)	70.8	45.7	21.5	Glucose syrup solids, lactose, sucrose	Malaysia
Danone Dumex	Dumex Mamil Gold Step 4	70.6	45.5	21.4	Lactose, glucose syrup solids, sucrose	Malaysia
Dutch Lady	Dutch Lady 123 (1+)	59.1	32.5	13.0	Glucose syrup solids, lactose, sucrose	Malaysia
Dutch Lady	Friso Gold Step 3 (1+)	63.0	33.2	12.2	Glucose syrup solids, sucrose	Malaysia
Dutch Lady	Dutch Lady 456 (3+)	61.9	32.4	13.0	Glucose syrup solids, lactose, sucrose	Malaysia
Dutch Lady	Friso Gold Step 4	62.3	34.7	14.8	Glucose syrup solids, sucrose	Malaysia
Fonterra Brands	Anmum Essential 3 (1+)	46.4	0	0	N/A	Indonesia
Fonterra Brands	Anmum Essential 4 (3+)	48.3	0	0	N/A	Indonesia
Fonterra Brands	Anmum Essential 3 (1+)	38.9	0	0	N/A	Malaysia
Fonterra Brands	Anmum Essential 4 (3+)	40.7	0	0	N/A	Malaysia
Frisian Flag	Frisian Flag 123 Vanilla (1+)	54.3	17.1	6.0	Sucrose, maltodextrin	Indonesia
Frisian Flag	Frisian Flag 456 Vanilla (3+)	51.4	11.4	4.0	Sucrose, maltodextrin	Indonesia
Indomilk	Biokids 1–3 Plain (1+)	57.5	25.0	10.0	Glucose syrup	Indonesia
Indomilk	Biokids 4–6 Plain (3+)	55.0	20.0	8.0	Glucose syrup	Indonesia
Mead Johnson	Enfagrow A+ Vanilla (1+)	65.0	32.5	13.0	Corn syrup solid, sucrose	Indonesia
Mead Johnson	Sustagen Junior 1+ Vanilla (1+)	70.0	37.5	15.0	Corn syrup solid, sucrose	Indonesia
Mead Johnson	Enfagrow A+ Step 3 (Original) (1+)	59.0	31.8	12.7	Corn syrup solids, lactose	Malaysia
Mead Johnson	Enfagrow A+ Step 3 (Vanilla) (1+)	58.0	30.8	12.3	Sucrose, corn syrup solids, lactose	Malaysia
Mead Johnson	Sustagen Junior 1+ (Vanilla) (1+)	71.0	43.8	17.5	Corn syrup solids, sucrose	Malaysia
Mead Johnson	Enfakid A+ Vanilla (3+)	65.0	30.0	12.1	Corn syrup solid, sucrose	Indonesia

Table S1. *Cont.*

Mead Johnson	Sustagen Kid 3+ Vanilla (3+)	67.5	32.5	13.0	Sucrose, corn syrup solid	Indonesia
Mead Johnson	Enfakid A+ Step 4 (Original)	61.0	31.5	12.6	Sucrose, lactose, corn syrup solids	Malaysia
Mead Johnson	Sustagen Kid 3+ (Vanilla)	68.0	38.5	15.4	Sucrose, corn syrup solids	Malaysia
Nestle	Dancow Regular 1+ Vanilla (1+)	53.3	10.0	3.0	Maltodextrin, sugar, lactose, honey	Indonesia
Nestle	Lactogen Gold (1+)	58.3	20.4	7.0	Lactose, maltodextrin, sugar	Indonesia
Nestle	Nutrigold 1+ (1+)	53.6	14.9	5.0	Sugar, maltodextrin, honey	Indonesia
Nestle	Dancow Batita (1+)	57.1	20.0	7.0	Sugar, maltodextrin, honey	Indonesia
Nestle	Nespray 1+ (1+)	52.5	22.2	8.0	Corn syrup solids, sucrose, lactose	Malaysia
Nestle	NESLAC - Excella Gold 1+ (Plain) (1+)	52.5	22.9	8.0	Corn syrup, sucrose, lactose	Malaysia
Nestle	Lactogen 3 (1–3 Years) (1+)	58.7	27.7	9.7	Lactose, maltodextrin, sucrose	Malaysia
Nestle	Nan 3 Pro (1–3 Years) (1+)	57.8	24.8	8.2	Lactose, maltodextrin	Malaysia
Nestle	Dancow Regular 3+ Vanilla (3+)	50.0	3.3	1.0	Sugar, maltodextrin, lactose, honey	Indonesia
Nestle	Dancow Datita Vanilla (3+)	60.0	20.0	7.0	Sugar, maltodextrin, honey	Indonesia
Nestle	Nespray 3+ (3+)	49.5	16.7	6.0	Corn syrup solids, sucrose, lactose	Malaysia
Nutricia	Nutrilon Royal 3 Vanilla (1+)	64.9	36.8	17.0	Glucose syrup solid, dextrose, lactose, fructose	Indonesia
Nutricia	Nutrilon Reguler 3 Vanilla (1+)	64.9	36.8	17.0	Glucose syrup solid, dextrose, lactose, fructose	Indonesia
Nutricia	Bebelac Complete (1+)	64.5	38.3	20.0	Glucose syrup solid, sucrose	Indonesia
Nutricia	Bebelac 3 Vanilla (1+)	64.9	36.8	17.0	Lactose, glucose syrup solid, sucrose	Indonesia
Nutricia	Nutrilon Royal 4 Vanilla (3+)	67.5	39.7	20.0	Glucose syrup solid, dextrose, fructose, lactose	Indonesia
Nutricia	Nutrilon Reguler 4 Vanilla (3+)	67.5	39.7	20.0	Glucose syrup solid, dextrose, fructose, lactose	Indonesia
Nutricia	Bebelac 4 Vanilla (3+)	66.5	38.3	19.0	Glucose syrup solid, lactose, sucrose, fructose	Indonesia
Sanghyang Perkasa	Chilkid Platinum Vanilla (1+)	59.4	18.8	6.0	Maltodextrin, sucrose, lactulose	Indonesia

Table S1. *Cont.*

Sanghyang Perkasa	Chilkid Regular Vanilla (1+)	59.4	18.8	6.0	Maltodextrin, sucrose, lactulose	Indonesia
Sanghyang Perkasa	Chil School Platinum Vanilla (3+)	65.2	34.8	16.0	Maltodextrin, sucrose, lactulose	Indonesia
Sanghyang Perkasa	Chil School Reguler Vanilla (3+)	65.2	34.8	16.0	Maltodextrin, sucrose, lactulose	Indonesia
Sari Husada	Vitalac 1+ Vanilla (1+)	54.3	17.1	6.0	Sucrose, lactose	Indonesia
Sari Husada	SGM Eksplor Presinutri Vanilla (1+)	60.0	22.9	8.0	Sucrose, lactose, maltodextrin, honey powder	Indonesia
Sari Husada	SGM Aktif Presinutri Vanilla (3+)	64.6	21.5	7.0	Sucrose, lactose, honey powder, dextrin, maltose	Indonesia
Snow Brand	Super Kid-Plus Step 3	52.7	16.8	5.5	Sucrose, lactose, dextrin	Malaysia
Wyeth	S26 Procal Gold Vanilla (1+)	61.4	31.8	14.0	Maltodextrin, lactose	Indonesia
Wyeth	Promise Gold Vanilla (3+)	60.4	34.0	18.0	Maltodextrin, lactose	Indonesia
Wyeth	Wyeth S-26 Promise Gold- Step 4 (Vanilla)	62.0	39.7	21.1	Maltodextrin, lactose	Malaysia
Wyeth	Wyeth S-26 Progress Gold Step 3 (Vanilla) (1+)	64.0	39.2	17.3	Maltodextrin, lactose	Malaysia

[1] Added carbohydrates were defined as the difference between the total carbohydrate content and the typical lactose content in whole milk powder, excluding fibre, the ingredient lists and nutrition information panels were used to calculate the percentage of declared carbohydrates coming from added sources, excluding dietary fiber ingredients (e.g., inulin); [2] Serving size specified by manufacturer on label; [3] Carbohydrate ingredients listed by manufacturer on label.
